# Hyperpyrexia: Its Causes, Prognosis, and Treatment

**Published:** 1907-03-09

**Authors:** 


### SPECIAL ARTICLE.

### HYPERPYREXIA: ITS CAUSES, PROGNOSIS, AND TREATMENT.

### 1. Definition.

The term hyperpyrexia signifies that the patient to whose case it is applied has a very high temperature, but for the exact point at which a temperature is high enough to merit the term there is no agreed standard. Some authors consider that a temperature of 105° F., or over, is hyperpyrexia; others do not apply the term unless the temperature is 106° F. or more. For the purposes of the present article the minimum level for hyperpyrexia will be regarded as 106° F.

### 2. The Commoner Causes of Hyperpyrexia in Great Britain.

Many series of cases of hyperpyrexia have been published at different times, but probably the cases occurring in a large London hospital will be most instructive to those engaged in general practice. Hence the following examples reported by Bryant from Guy's Hospital may be usefully noted:

| Disease. | Cases. | Deaths. | No. of Cases of Hyper-pyrexia. | Deaths. |
|---|---|---|---|---|
| 1. Brain lesions :— | | | | |
| (a) Fractured skull ... | 10 | 10 | 22 | 22 |
| (b) Meningitis (tuberculous or suppurative) | 6 | 6 | | |
| (c) Cerebral hæmorrhage | 2 | 2 | | |
| (d) Cerebral abscess ... | 2 | 2 | | |
| (e) Cerebral softening ... | 1 | 1 | | |
| (f) Cerebral cyst ... ... | 1 | 1 | | |
| 2. Pyæmia or septicæmia ... ... ... | | | 17 | 15 |
| 3. Spinal cord lesions :— | | | | |
| (a) Fractured cervical spine and cord injury | 6 | 6 | 7 | 6 |
| (b) Myelitis ... ... | 1 | 0 | | |
| 4. Acute rheumatism ... ... ... ... | | | 7 | 4 |
| 5. Typhoid fever ... ... ... ... | | | 6 | 3 |
| 6. Scalds and burns ... ... ... ... | | | 6 | 5 |
| 7. Lobar pneumonia ... ... ... ... | | | 5 | 3 |
| 8. Stricture of urethar; ascending nephritis | | | 4 | 2 |
| 9. Malignant disease ... ... ... ... | | | 4 | 4 |
| 10. Broncho-pneumonia ... ... ... | | | 4 | 4 |
| 11. Fungating endocarditis ... ... ... | | | 3 | 3 |
| 12. Delirium tremens ... ... ... ... | | | 3 | 3 |
| 13. Tuberculosis ... ... ... ... | | | 2 | 2 |
| 14. Tetanus ... ... ... ... ... | | | 2 | 2 |
| 15. Heat stroke ... ... ... ... ... | | | 2 | 1 |
| 16. Children without p.-m. evidence of disease ... ... ... ... ... | | | 2 | 2 |
| 17. Erysipelas ... ... ... ... ... | | | 1 | 1 |
| 18. Severe injury (fractured pelvis and liver) ... ... ... ... | | | 1 | 1 |
| 19. Syphilis without obvious brain lesion ... | | | 1 | 1 |
| 20. Hysteria ... ... ... ... ... | | | 1 | 0 |
| | | | 100 | 84 |

This list in itself provides a considerable number of causes of hyperpyrexia, but there are other diseases in which the condition has also occurred in this country, namely, chorea, scarlet fever, small-pox, typhus, epidemic cerebro-spinal meningitis, after labour, epilepsy, exophthalmic goitre, and influenza; whilst abroad hyperpyrexia has additional causes in cholera, malaria, dengue, intermittent fever, glanders, Malta fever, and yellow fever. If the standard of hyperpyrexia were fixed at 105° F., instead of 106° F., the list of causes would not be very different, but the total number of cases and the relative incidence in the different diseases would be somewhat altered.

### 3. The Likelihood or the Reverse, that Patients Suffering from the Above Diseases will Develop Hyperpyrexia.

The figures as given above merely give the total number of cases of each disease in which hyperpyrexia occurred. In order to know what likelihood there is of the temperature going to 106° F. or upwards in any particular disease, we must also know what proportion of patients suffering from such disease have been observed to develop hyperpyrexia. This is illustrated by the following figures taken from the same source as the above:—

| | Total Number of Cases with or without Hyperpyrexia. | Percentage in which Hyperpyrexia occurred. |
|---|---|---|
| Heat stroke ... ... | 11 | 18.0 |
| Fractured cervical spine | 29 | 17.2 |
| Tetanus ... ... ... ... | 24 | 8.0 |
| Pyæmia or septicæmia ... | 246 | 7.3 |
| Cerebral abscess ... ... | 32 | 6.2 |
| Meningitis (tuberculous or suppurative) ... | 264 | 3.4 |
| Fungating endocarditis | 115 | 2.6 |
| Delirium tremens ... | 135 | 2.2 |
| Fractured skull ... | 458 | 2.2 |
| Typhoid fever ... ... | 608 | 1.0 |
| Burns and scalds ... | 803 | 0.8 |
| Broncho-pneumonia ... | 641 | 0.6 |
| Lobar pneumonia ... | 1196 | 0.4 |
| Acute rheumatism ... | 1821 | 0.38 |
| Erysipelas ... ... | 410 | 0.24 |

It will be seen that in none of the above mentioned conditions is it the rule to get hyperpyrexia; that the liability to it is far greater in heat stroke and in fractured cervical spine than in any other affection; and that in typhoid fever, burns, and scalds, broncho-pneumonia, lobar pneumonia, acute rheumatism, and erysipelas, it is not one case in a hundred that has a temperature of 106° F. The importance of stating this lies in the fact that medical students are extremely liable to pick up erroneous ideas upon the subject, particularly in connection with rheumatic fever. In examinations it not infrequently happens that hyperpyrexia is given as one of the most important complications of this disease, and it is even sometimes described as common.

### 4. The Influence of Season upon Occurrence of Hyperpyrexia.

All writers upon the subject are agreed that hyperpyrexia is more common in hot weather than in cold. This is perhaps not surprising, seeing that the loss of heat from the body by radiation and convection is much greater when the surroundings are cold; but when one remembers that the temperature of the human body in health is practically constant, whether in the Tropics or at the Poles, it would be difficult to conclude on purely *a priori* grounds what the influence of the time of year upon the pyrexia would be. The facts are brought out very clearly indeed by Bryant's table, which is as follows:

| Month of the Year. | No. of Cases of Hyperpyrexia. |
|---|---|
| January | 5 |
| February | 8 |
| March | 2 |
| April | 5 |
| May | 7 |
| June | 10 |
| July | 11 |
| August | 20 |
| September | 10 |
| October | 9 |
| November | 7 |
| December | 6 |
| | 100 |

One-fifth of all his cases occurred in August, and over one-half of the hundred cases were observed during the months June to September. This suggests that a potent factor in the production of hyperpyrexia is a difficulty in getting rid of the heat produced; and this is a strong argument in favour of treating the condition by sponging, cold baths, and so on. These will be briefly discussed in the later part of this article.

5. GENERAL REMARKS UPON HYPERPYREXIA IN THE MORE IMPORTANT CONDITIONS UNDER WHICH IT OCCURS.

(*a*) *Brain lesions*: It is noteworthy that practically every case in which a brain lesion leads to hyperpyrexia is fatal. To know this for meningitis is not so important, because a fatal termination, apart from hyperpyrexia, is so common; but it is a useful fact to remember in cases of cerebral hæmorrhage and of fractures of the skull. It is also useful to know that cerebral embolism and cerebral thrombosis are not associated with hyperpyrexia. The hæmorrhage which is most liable to cause a very high temperature is one into the pons; but, as Hale White showed, any lesion which suddenly interferes with the corpus striatum, centrum ovale, or optic thalamus, may produce a similar result. It is believed that this is because there is a heat-regulating centre somewhere in this region, and that the hyperpyrexia is due to a disturbance of the normal balance between heat production and heat loss. The temperature may be as high as 110° F., and, seeing that the lesion is itself fatal in all these cases, treatment of the hyperpyrexia is of little avail.

(*b*) *Spinal lesions*: Fractures of the spine elsewhere than in the cervical region do not cause hyperpyrexia. Fractures in the cervical region, on the other hand, do so in a considerable proportion of cases, and all such cases die. It seems likely that there is a subsidiary heat-regulating centre in the cervical spinal cord, and that whether or not hyperpyrexia will result from the fracture depends upon the extent to which the cord is injured. If the injury is sufficient to cause hyperpyrexia, treatment of the latter means useless disturbance of the patient, because the injury is in itself fatal.

(*c*) *Acute rheumatism*: As has been already pointed out, hyperpyrexia in rheumatic fever is proportionately very rare; nevertheless acute rheumatism is so common a disease that sooner or later every practitioner is likely to come across a case associated with very high temperature. The noteworthy features are that rheumatic hyperpyrexia occurs in females more often than it does in males, the proportion being 2 to 1; that it is almost unknown under ten years of age; that it is much more likely to occur between the ages of 20 and 30 than at any other age; that it is very much more common in first attacks of rheumatic fever than it is in later ones (67 per cent. of the cases are in first attacks); that it is much more likely to occur during the second week of the illness than before or after this; that over half the cases occur during the four summer months; that delirium is an almost certain accompaniment or precursor of the hyperpyrexia; that the prognosis is not so bad as it is in some other examples of hyperpyrexia, seeing that nearly half the cases recover; that there are no special post-mortem changes to account for the pyrexia, so that apparently the hyperpyrexia is itself the cause of death in the fatal cases; and that, therefore, treatment should be active in combating the high temperature itself. The only useful method of treatment is the early application of cold to the exterior of the body by one or other of the methods discussed below.

(*d*) *Stricture of the Urethra and Catheterisation*: Hyperpyrexia in these conditions may occur as the result of suppurative ascending nephritis, in which case the prognosis is absolutely bad; on the other hand it may occur without any evidence of nephritis, especially as a sequela of catheterisation. How the hyperpyrexia is produced is uncertain; some think it is due partly to a reflex effect upon the heat-regulating centres; it seems more likely, however, that the catheterisation abrades the urethral mucosa sufficiently to allow absorption of a large amount of microbial poisons, and that it is these which cause the pyrexial reaction. It is common to find rigors and delirium at the same time. It is noteworthy that out of four cases two recovered; so that, bad though the prognosis is, it might be worse. We have little or no means of telling which cases are dying of suppurative nephritis, and which are merely suffering from toxic absorption from the site of stricture; it is therefore most important to treat all these cases as though they could recover, and therefore to relieve the temperature by the use of cold baths and so on.

(*e*) *Lobar Pneumonia and Broncho-pneumonia*: In all the four cases of broncho-pneumonia in Bryant's list, hyperpyrexia was followed by death. This probably overstates the risk in these cases; temperatures of 105° F. in children with broncho-pneumonia are not uncommonly followed by recovery, and it is not impossible for recovery to follow temperatures of 106° F. or even over this. The body temperature is far less stable in children than in adults, and as a rule high temperatures are of proportionately less significance. Nevertheless the high mortality recorded by Bryant in broncho-pneumonia indicates the necessity for bringing down the temperature when it reaches this height. It is probably best to begin sponging when the temperature is not yet 105° F., in order to prevent it going higher. In lobar pneumonia, on the other hand, a temperature of 105° F. is comparatively common, and does not necessarily mean a bad prognosis. In Bryant's cases the temperature was 106.4° F. in two of the five cases, and both these recovered; in the other three it was 107.8° F., 108.2° F., and 108.2° F. respectively, and all these died. Hyperpyrexia, if it does not exceed 106° F., therefore, does not render the prognosis very bad in lobar-pneumonia; but if the temperature goes above 106.6° F. the patient is almost certain to die. Moderately high and continued fever is a much better sign upon the whole than is a low or irregular temperature; and since the illness is likely to be short, there is less necessity to treat the pyrexia itself in this disease than in almost any other. It is usually quite safe to leave matters alone if the temperature does not exceed 105° F., but above this it is better to resort to tepid sponging, whilst some authorities even go so far as to recommend rubbing with block ice. One benefit of the sponging will almost always be that the patient will sleep after it, and of all things sleep is probably the most beneficial in the relief of lobar-pneumonia.

(*f*) *Typhoid Fever*: Hyperpyrexia in this disease has not so extremely bad a prognosis as one might think. Half of the patients in Bryant's table recovered, though the temperature of one of these reached 106.8° F. No case with a temperature higher than this got well; but it seems clear that temperatures no higher than 106° F., serious though they almost always are, are not proportionately so serious in typhoid fever as they are in many other conditions. Nevertheless, as the fever is likely to continue long, and the heart-muscle is bound to suffer if it is continuously bathed in fluid at a high temperature, the modern treatment is directed towards preventing the fever exceeding even 103.5° F. if possible. It is the general rule to start cold baths or sponging in enterica when the thermometer reaches 103.5° F., and to continue these at intervals so long as this temperature is maintained. The results of this plan of treatment have been most encouraging.

(*g*) *Erysipelas, Pyæmia, and Septicæmia*: Hyperpyrexia is remarkably rare in erysipelas, notwithstanding the fact that considerable pyrexia is the rule. In pyæmia and septicæmia, on the other hand, one case in fourteen has hyperpyrexia, usually associated with rigors. The prognosis in these conditions is naturally bad even without hyperpyrexia, but recovery is possible, even though a temperature of 106.4° F. be reached; above this a fatal issue is practically certain. It is usually impossible during life to say whether the patient's condition is pyæmia or septicæmia; if the patient recovers, it is usually called pyæmia; if he dies, septicæmia. Hence, since it is so difficult to distinguish the two, all must be treated as recoverable cases, and therefore, since hyperpyrexia itself adds to the gravity of the condition, the hyperpyrexia must in all cases be treated by cold applications in addition to serum and other treatment for the septic state.

(*h*) *Delirium tremens*: Hyperpyrexia in this is always fatal; this fact reminds us that there is a rare disease called acute delirious mania, which is usually classed amongst the insanities. Acute delirious mania has an unknown pathology; it is always associated with high fever and is always fatal; one wonders what difference there is between acute delirious mania and delirium tremens with hyperpyrexia.

(*i*) *Heat Stroke*: The majority of cases of heat stroke occurring in England do not suffer from extreme fever; in the tropics the fever is much more marked. It not only reaches 106° F., but may go much higher, even to 110° F. or more. There are at least two points of particular interest in this connection. The first is that the symptoms of this form of heat stroke are very similar to those of pontine hæmorrhage, seeing that in both the patient is comatose, with flaccid limbs, stertorous breathing, contracted pupils, and pyrexia; the second is that a considerable proportion of the cases of heat stroke recover even though the temperature has exceeded 107° F. In heat stroke the obvious treatment is by applications of cold to the head and body—hosing with cold water in the tropics; it is therefore also right, if the diagnosis be undecided between pontine hæmorrhage in the summer months and heat stroke, to give the patient the benefit of the doubt. Treat the hyperpyrexia actively as though it were a case of heat stroke; for this will give the best chance of recovery if it be the latter, whilst if it be a pontine hæmorrhage with hyperpyrexia the ending must be fatal whether anything or nothing be done.

(*j*) *Hysterical*: Extraordinary temperatures have been recorded under this head, ranging from 106° F. to 151° F., and not followed by death. In all such cases there is ample room for scepticism, and not a few extraordinary temperatures have been brought about artificially by malingerers. Some undoubted malingerers have learned some knack of rubbing the bulb of the thermometer either with the tongue or fingers, sending up the temperature of the mercury by friction. Others learn that if a thermometer be wrapped in the edge of the blanket and then held in the axilla or in the mouth the mercury can be sent up to a great height, owing to the heat produced by some physical force connected with capillary attraction when the sweat or saliva enters the pores and threads of the blanket. Even, however, when all such cases are eliminated, the physician still has to reckon with very high temperatures recorded by the thermometer in some patients, even when he takes the temperature himself in the usual way. The patients are practically all women, young women of nervous temperament. They use no trick, yet in some way unknown either to patient or doctor the thermometer goes up absurdly high, though there may be nothing really the matter with the patient. It can scarcely be supposed that the blood itself ever reaches a temperature of 120° F., for example, and yet in different well-authenticated cases the temperature, taken without fraud, has reached 131° F., 128° F., 127° F., 122° F., 120.8° F., 117° F., 116.4° F., 115.8° F., 115.8° F., 113° F., 111.6° F., 111° F., 111° F., 108.2° F., and in a great many other cases over 106° F. So remarkable does this appear that anyone who has not actually met with such a case is certain to be sceptical. That the temperatures are not fraudulent, but real, in the sense that they are registered by good thermometers used in the ordinary way, is proved beyond doubt. In many cases the high temperatures have been registered by three thermometers simultaneously—one in the mouth and one in each axilla. In Galbraith's case, to prevent fraud, the patient was stripped completely and placed in a chair; the mouth and axillæ were carefully examined, and the doctor himself took the temperature in mouth and axillæ, watching the patient all the time, and taking precautions to see that the thermometer was not tipped nor in any way interfered with; the thermometer in the mouth registered 131° F., that in the axilla 137° F.

We have already said that these high temperatures cannot conceivably be those of the blood. One supposes that some occult chemical or electro-chemical changes must be taking place in the fluids which bathe the bulbs of the thermometers, or something of that kind. We do not really know anything definite about this form of hyperpyrexia except that it does occur, and therefore must be reckoned with in differential diagnosis. A short while since a hospital nurse in Surrey "had a temperature" of 110° F. on two successive days, and a consultant was sent for to decide what it meant, deep-seated suppuration being suspected. The temperature was real, in so far as the thermometer could not help registering it, but it was unreal as regards the patient's body. She was not ill at all. This fact—that the patients are young nervous women who are not extremely ill though the thermometer registers hyperpyrexia—gives the main point in differential diagnosis; and as regards treatment, this is the only form of hyperpyrexia in which no particular treatment is required. Cold sponging, and so on, is not contra-indicated, for it will do no harm; but the patients recover without treatment if the diagnosis of hysterical hyperpyrexia be correct.

